# Lithium Toxicity With Lasting Mental Status Impairment

**DOI:** 10.7759/cureus.28076

**Published:** 2022-08-16

**Authors:** Rachel M Fenner, Samantha Bookbinder, Joseph Kratzer, Ashley McNeal

**Affiliations:** 1 Endocrinology and Diabetes, AnMed Health, Anderson, USA; 2 Neurology, Medical University of South Carolina, Charleston, USA; 3 Neurology, AnMed Health, Anderson, USA; 4 Family Medicine, AnMed Health, Anderson, USA

**Keywords:** chorea in lithium toxicity, cognitive side effects, lithium, lithium poisoning, lithium toxicity

## Abstract

A 49-year-old female taking lithium for bipolar affective disorder for over 20 years presented with lithium toxicity resulting in declining mentation. Lithium poisoning has been well documented to cause acute gastrointestinal, cardiac, and neurological side effects, along with long-term neurologic sequelae. There has, however, been scant discussion on the potential long-term effects on mentation. The following case report illustrates a possible example.

## Introduction

Lithium poisoning has characteristic acute symptoms as well as a long-term syndrome of irreversible lithium-effectuated neurotoxicity (SILENT)[1], but possible persistent cognitive impairment has not been widely discussed. A patient who presented with a lithium serum level of 3.3 was found to have progressively declining mental status for months after discharge. A 49-year-old female, with a past medical history of breast cancer, hypothyroidism, sarcoidosis, bipolar affective disorder, prior coronavirus disease 2019 (COVID-19) infection, and Type 2 Diabetes Mellitus, who was taking lithium for over 20 years, presented with lithium toxicity resulting in lasting, declining mentation.

## Case presentation

A 49-year-old female, with a history of breast cancer, hypothyroidism, sarcoidosis, bipolar affective disorder, prior coronavirus disease 2019 (COVID-19) infection, and Type 2 Diabetes Mellitus, presented to the emergency room (ER) complaining of confusion. She stated that she had vomiting, diarrhea, and headaches for three weeks. She fell and hit her head the night before presenting to the ER, at which time her blood sugar was noted to be 47. After her fall, she called her mother, who reported that the patient appeared confused. The patient and her mother denied any loss of consciousness, headache, or neck pain. There was no reported history of alcohol or substance abuse, nor any family history of schizophrenia or early dementia. 

When she arrived in the ER, she exhibited confusion and short-term memory difficulties. Abnormal involuntary movements including myoclonus and chorea were noted, as well as ataxia. Her blood glucose was 131 and her blood alcohol was negative. She had mild hyponatremia (sodium 133 mg/dL) and elevated creatinine (1.31 mg/dL). Acetaminophen levels and salicylate levels were normal.

Further investigation revealed that she had been taking lithium for bipolar affective disorder for over 20 years. Her bipolar affective disorder had been well controlled on lithium throughout that time. Her lithium level was found to be elevated at 3.3. Her pill count was accurate, without evidence of incorrect self-dosing, so acute overdose was not suspected. Reviewing her prior labs showed baseline creatinine two months prior of 0.90 mg/dL. 

The patient was admitted to the ICU with a neurology consult, and an internal jugular vein dialysis catheter was placed. After one treatment of hemodialysis lasting two hours, her lithium level dropped to 1.2, but she continued to exhibit myoclonus. Further workup included a head CT on the first day of her admission and a head MRI on the fifth day of admission to rule out stroke or other intracranial diseases. She was given benztropine to control myoclonus and discharged home. Her bipolar affective disorder is to be treated with lamotrigine.

After discharge, she continued to have worsening memory problems (Montreal Cognitive Assessment {MoCA} 7/30 and Mini-Mental Status Exam {MMSE} 14) and myoclonus. Four months after her hospitalization, her condition continued to worsen with short-term memory loss, sundowning, and difficulty caring for herself. She was fully dependent on a caretaker and unable to live alone.

## Discussion

The syndrome of irreversible lithium effectuated neurotoxicity (SILENT) is characterized by cerebellar dysfunction, extrapyramidal symptoms, brainstem dysfunction, and dementia. Other symptoms can include nystagmus, choreoathetoid movements, myopathy, and blindness. A case report expanded the possible symptoms to include visual and auditory perceptual changes [2]. The patient described in the case report has a significant lasting neurologic and motor sequela after an acute toxicity event with a lithium level recorded as 2.6 mmol/L. Additionally, they showed a temporary decline in Montreal Cognitive Assessment and lasting “deficits in memory, executive function, handwriting”. In a different study discussing three cases, cognitive impairment has been reported due to lithium toxicity [3]. However, this cohort was a population taking additional medications which can contribute to impaired cognition, including benztropine and high-dose haloperidol. Additionally, these individuals experienced complications sub-acutely after initiation of lithium therapy.

Senga et al. reported a similar outcome in their own case study, in which a war veteran presented in the emergency department (ED) from lithium toxicity with a recorded level of 2.8 mEq/L [4]. The 56-year-old male had been taking lithium chronically as prescribed for post-traumatic stress disorder and bipolar disorder and presented to the ED with altered mental status and seizures associated with elevated lithium levels and renal insufficiency. Despite clearing the agent and treatment with antiepileptics, repeated EEGs after two months continued to show a generalized slowing; and the patient only regained only non-purposeful limb movements and the ability to open one eye. Another similar case was reported by Vallianou et al., in which a 74-year-old male who had taken lithium chronically for 37 years and was admitted to the hospital for dysarthria, agitation, and confusion for five days [5]. Initial labs showed a serum lithium level of 2.03 mEq/L. Despite a decrease in lithium levels, the patient continued to have ataxia, memory deficits, nystagmus, and extrapyramidal symptoms and was diagnosed with SILENT syndrome. However, the patient’s agitation and confusion passed after lithium levels completely normalized, and the memory deficits and extrapyramidal signs improved after one month.

## Conclusions

All three of cases discussed are similar in that neurologic sequelae persisted despite aggressive treatment and normalization of lithium levels. However, the actual presentation of the patient, the timeline, and the extent of the symptoms varied widely. Thus, there needs to be more investigation done to determine the full breadth of possible presentations and outcomes so that clinicians may be more aware of the varied presentations and time courses.
